# Peripheral blood HIV-1 DNA dynamics in antiretroviral-treated HIV/HCV co-infected patients receiving directly-acting antivirals

**DOI:** 10.1371/journal.pone.0187095

**Published:** 2017-10-27

**Authors:** Gabriella Rozera, Gabriele Fabbri, Patrizia Lorenzini, Ilaria Mastrorosa, Laura Timelli, Mauro Zaccarelli, Alessandra Amendola, Alessandra Vergori, Maria Maddalena Plazzi, Stefania Cicalini, Andrea Antinori, Maria Rosaria Capobianchi, Isabella Abbate, Adriana Ammassari

**Affiliations:** 1 Laboratory of Virology, National Institute for Infectious Diseases “L. Spallanzani” IRCCS, Rome, Italy; 2 Clinical Department, National Institute for Infectious Diseases “L. Spallanzani” IRCCS, Rome, Italy; Rush University, UNITED STATES

## Abstract

**Background:**

Aim was to determine the dynamics of peripheral blood mononuclear cells (PBMC)- associated total HIV-1 DNA in successfully ART-treated HIV/HCV co-infected patients receiving DAA treatment and to explore possible virological hypotheses underlying the phenomenon.

**Methods:**

Longitudinal, single-centre study measuring total HIV-1 DNA before the start of DAA, at the end of treatment (EOT), and 3 months after treatment. Univariable and multivariable analyses were used to assess factors associated with HIV-1 DNA increase ≥0.5 Log copies/million PBMC. Episomal 2-LTR forms, residual HIV-1 viremia and proviral DNA quasispecies evolution were also investigated.

**Results:**

119 successfully ART-treated HIV/HCV co-infected patients were included. Median baseline HIV-1 DNA was 3.84 Log copies/million PBMC (95%CI 3.49–4.05), and no significant variation with respect to baseline was found at EOT and after 3 months of DAA termination. In 17% of cases an increase ≥0.5 Log copies/million PBMC was observed at EOT compared to baseline. HIV-1 DNA increase was independently associated with lower baseline HIV-1 DNA, longer HIV suppression, raltegravir-based ART and previous exposure to interferon/ribavirin for HCV treatment. In none of the patients with HIV-1 DNA increase, 2-LTR forms were detected at baseline, while in 2 cases 2-LTR forms were found at EOT, without association with residual HIV-1 RNA viremia. No evidence of viral evolution was observed.

**Conclusions:**

In successfully ART-treated HIV/HCV co-infected patients receiving DAA, PBMC-associated total HIV-1 DNA was quite stable over time, but some patients showed a considerable increase at EOT when compared to baseline. A significantly higher risk of HIV DNA increase was found, in presence of lower cellular HIV reservoir at baseline. Activation of replicative-competent virus generating new rounds of viral replication seems unlikely, while mobilization of cell-associated HIV from tissue reservoirs could be hypothesized.

## Introduction

The pool of latently HIV-infected cells in long-term antiretroviral therapy (ART)-treated patients is quite stable over time and represents the main obstacle for HIV eradication [1–5]. HIV-1 DNA levels in the peripheral blood have well been described in ART-treated and untreated chronic and primary HIV-1 infection [6–12], but little information is available about HIV reservoirs in the HIV/HCV co-infected population in general and during HCV treatment. Some studies have investigated whether interferon-based therapy in HIV mono-infected and HIV/HCV co-infected persons affects HIV-1 expression and CD4 T-cell activation, and concluded that interferon may play a role in decreasing integrated and episomal circular forms of HIV-1 DNA, cell-associated HIV-1 RNA, as well as immune activation [13–18]. In contrast, an increase of HIV-1 DNA levels after directly-acting antivirals (DAA) treatment was found in a recent longitudinal study on the time course of cellular HIV-1 DNA in a small population of HIV/HCV co-infected patients [19].

The sharing of the same metabolic pathway by DAA and antiretrovirals makes the scenario even more complex [20]. In fact, it is often necessary to modify well-established ART regimens by switching from boosted protease inhibitors or first-line non-nucleoside reverse transcriptase inhibitors to integrase strand transfer inhibitor (INSTI)-based treatments, which are characterized by a more favourable drug-to-drug interaction profile [21–22]. To date, the virological consequences of this strategy are not completely understood and may be underestimated, because contradictory evidence is available about episomal HIV-1 DNA levels after switch to this class of antiretrovirals [23–25].

Aim of this longitudinal study was to determine the dynamics of peripheral blood mononuclear cells (PBMC)-associated total HIV-1 DNA in successfully ART-treated HIV/HCV co-infected patients receiving DAA treatment and to explore possible virological hypotheses underlying the phenomenon.

## Materials and methods

### Study design

This longitudinal, single-centre study was approved by the local Ethics Committee of the INMI “L. Spallanzani”, Rome, Italy, and ART-treated patients with HIV/HCV co-infection were included after signing the informed consent. Peripheral blood samples were collected before the start of DAA (T0), at 1 month of DAA, at the end of treatment (EOT) (either at completion of 12 or 24 weeks of DAA), and after 3 months of DAA termination. The caring physician prescribed DAA: a) based on International guidelines [26] and access to HCV treatment based on the indication of the Italian Medicines Agency for patients with F3-F4 fibrosis or HCV-associated extrahepatic disorders; b) within an expanded access to ombitasvir/paritaprevir/ritonavir with or without dasabuvir for patients with F2 hepatic fibrosis. Sustained virological response to DAA was defined by HCV RNA <12 UI/mL after 3 months of DAA termination (SVR12).

Inclusion criteria for patients in the study were to have: i) HIV-1 RNA<40 copies/mL at T0; ii) HIV-1 DNA measurement before DAA initiation (T0) and at least at one follow-up time-point. Based on HIV-1 RNA suppression over time, patients were defined “optimal virological controllers” (OVC), if they maintained complete virological suppression during the entire observation period (i.e. HIV-1 RNA undetectable <40 copies/mL in all samples), and “sub-optimal virological controllers” (SVC), if HIV-1 RNA was detectable <40 copies/mL or >40 copies/mL in at least one follow-up sample.

### Laboratory investigations

Plasma HIV-1 RNA was measured by a commercially available kit (Abbott RealTime HIV-1 assay, Abbott Park, Illinois, U.S.A) with a limit of detection (LOD) of 40 copies/mL and by a modified protocol of the same method to reach 5 copies/mL as LOD for residual HIV-1 viremia [27]. HCV RNA was quantified in plasma with the Abbott RealTime HCV assay with a LOD of 12 IU/mL.

Total HIV-1 DNA was extracted from PBMC by QIAsymphony DNA Midi Kit (QIAGEN, S.r.l. Milan, Italy) and quantified by real-time PCR targeting LTR region. DNA was amplified with the sense primer NEC 152 (GCCTCAATAAAGCTTGCCTTGA) and the reverse primer NEC 131 (GGCGCCACTGCTAGAGATTTT) in the presence of a dually (FAM and TAMRA) labelled NEC LTR probe (AAGTAGTGTGTGCCCGTCTGTTRTKTGACT). As standard curve, dilutions of 8E5 cell DNA containing 1 proviral copy per cell were used [7]. Episomal unintegrated HIV-1 DNA was quantified by a specific PCR targeting a LTR region specific for 2-LTR circular forms [28, 29]. An additional real-time PCR targeting the housekeeping cellular hTERT gene was used to refer total HIV-1 DNA and 2-LTR copies to the amount of the cells present in the analysed samples [30]. All the in house real-time PCR have a LOD of 3 copies/reaction volume. The amount of 2-LTR forms was indicated as a fraction of total HIV-1 DNA.

Viral diversity of PBMC-associated proviral HIV-1 DNA quasispecies was performed by limiting-dilution PCR as in [31]. In particular, a portion of the *env* gene coding for C2-V5 regions of viral glycoprotein 120 was amplified by nested PCR using the outer sense primer ATGGGATCAAAGCCTAAAGCCATGTG (position 6557–6582 in HXB2), the outer antisense primer AGTGCTTCCTGCTGCTCCCAAGAACCCAAG (position 7822–7792 in HXB2), the inner sense primer CAGCACAGTACAATGTACACA (position 7002–7021 in HXB2) and the inner antisense primer CTTCTCCAATTGTCCCTCA (position 7648–7666 in HXB2), using a proof-reading DNA polymerase (Platinum Taq DNA Polymerase High Fidelity, Thermo Fisher Scientific, Milan, Italy). For each infected subject, on average 7 (range, 4–7) sequences were obtained at the different time points before, during and after DAA treatment. All the amplicons were sequenced using the BigDye1.1 terminator kit (Applied Biosystems, Foster City, CA) following the manufacturer’s instructions, with the same primers used in the second round of nested PCR and an ABI310 automatic sequencer. Nucleotide sequences were aligned with the CLUSTAL W program, using BioEdit Software (version 7.0.5.3). Genetic heterogeneity (diversity, i.e. mean number of nucleotide substitutions/site) of the viral quasispecies was established using Kimura two-parameter substitution model by MEGA software package (Version 6.0). Relationships among the variants present at baseline and during DAA were evaluated constructing a maximum likelihood (ML) phylogenetic tree by MEGA program. Bootstrap values >80% were considered significant.

### Statistical analysis

Results of HIV-1 DNA measurements and of continuous variables were summarized as the median value with the interquartile range (IQR). Categorical variables were specified as absolute number and frequency. To avoid regression to the mean, the mean of HIV-1 DNA measurements obtained before the start of DAA (T0) and at 1 month of DAA (T1) was used as baseline value. The change in median HIV-1 DNA between measurements at EOT or 3 months after DAA and baseline was calculated and expressed as a positive number in presence of an increase and as a negative number if a decrease was found. Comparison between median values at baseline and EOT or 3 months after DAA were carried out using paired Wilcoxon test. Chi Square or Fisher’s exact test were employed to compare categorical variable when appropriate.

Factors associated with the increase of total HIV-1 DNA in PBMC >0.5 Log at EOT were analysed using univariable and multivariable logistic regression and expressed by odds ratio (OR) and 95% confidence interval (95%CI). The multivariable regression model included all variables with a p-value <0.05 at univariable exploration. The Pearson correlation coefficient was used to assess the correlation between baseline HIV-1 DNA levels and its absolute increase between baseline and EOT. All p-values were two-tailed and if the result was <0.05 it was considered as statistically significant. Statistical analysis was performed with Stata 10.1.

## Results

### Patient population

A total of 119 HIV/HCV co-infected patients were included. General characteristics as well as HIV- and HCV-related variables are shown in Table 1. Most patients were male gender (79%), and overall median age was 53 years (IQR, 50–56). All patients were receiving ART and median time of HIV suppression was 8.9 years (IQR, 3.2–14.0). ART regimen during DAA included INSTI in 37 patients (31.1%), and in 29 (24.4%) cases raltegravir was prescribed. In all patients raltegravir was in place for several months before the start of DAA. DAA treatment was administered for 12 or 24 weeks in 50.4% and 49.6% of cases, respectively.

**Table 1:** Characteristics at study inclusion of the 119 patients.

General characteristics and demographics	
Male gender, n (%)	94 (79.0)
Age, median years (IQR)	53 (50–56)
**HIV-related variables**	
HIV transmission mode, n (%)	
- heterosexual	14 (11.8)
- MSM	5 (4.2)
- IVDU	91 (76.4)
- other/unknown	9 (7.6)
Years of known HIV infection, median (IQR)	24 (18–29)
Years of HIV suppression, median (IQR)	8.9 (3.2–14.0)
HIV-1 RNA, n (%)	
- detectable <40 copies/mL	26 (21.8)
- undetectable <40 copies/mL	93 (78.2)
Total HIV-1 DNA, median log copies/million PBMC (IQR)	3.84 (3.49–4.05)
Total lymphocyte, median cells/mmc (IQR)	1900 (1400–2500)
CD4 cell count, median cells/mmc (IQR)	549 (340–752)
CD4/CD8, median (IQR)	0.74 (0.51–1.02)
Change of ART before DAA, n (%)	38 (31.9)
Type of ART during DAA, n (%)	
- NNRTI + 2NRTIs	32 (26.9)
- PI/b + 2NRTIs	33 (27.7)
- INSTI + 2NRTIs	23 (19.3)
- INSTI + PI/b	9 (7.6)
- PI/b monotherapy	8 (6.7)
- INSTI + NNRTI	5 (4.2)
- PI/b + NNRTI	3 (2.5)
- other	6 (5.0)
Raltegravir-including ART, n (%)	29 (24.4)
**HCV-related variables**	
Baseline Log_10_ HCV RNA, median (IQR)	5.9 (5.2–6.4)
HCV genotype, n (%)	
- 1 / 1a / 1b	76 (63.9)
- 2a / 2c	-
- 3 / 3a	28 (23.5)
- 4 / 4c / 4d	15 (12.6)
Fibrosis staging, n (%)	
- F1/F2	21 (17.6)
- F3	39 (32.8)
- F4	59 (49.6)
IL28B subtype, n (%)	
- CC	12 (10.1)
- CT	25 (21.0)
- TT	13 (10.9)
- unknown	69 (58.0)
Previous IFN/RBV treatment, n (%)	35 (29.4)
Type of DAA treatment, n (%)	
- sofosbuvir-based +/- RBV	88 (73.9)
- ombitasvir/paritaprevir/ritonavir+/-dasabuvir +/- RBV	31 (26.1)
DAA duration, n (%)	
- 12 weeks	60 (50.4)
- 24 weeks	59 (49.6)

MSM = men having sex with men; IVDU = intravenous drug use; DAA = directly-acting antivirals; ART = antiretroviral therapy; NRTI = nucleoside/nucleotide inhibitor; NNRTI = non nucleoside transcriptase inhibitor; PI = protease inhibitor; INSTI = Integrase Strand Transfer Inhibitor; IFN = interferon; RBV = ribavirin.

At the time of the analysis, 97 patients had reached EOT and 84 of them were evaluated at 3 months after completion of DAA: SVR12 was achieved in 78 of patients (92.9%), while HCV relapse was observed in 6 cases. Blood samples included in the analyses were: 119 at T0, 112 at T1, 97 at EOT, and 84 at 3 months after DAA termination.

### Peripheral blood PBMC-associated total HIV-1 DNA and plasma HIV-1 RNA

**Fig 1:**
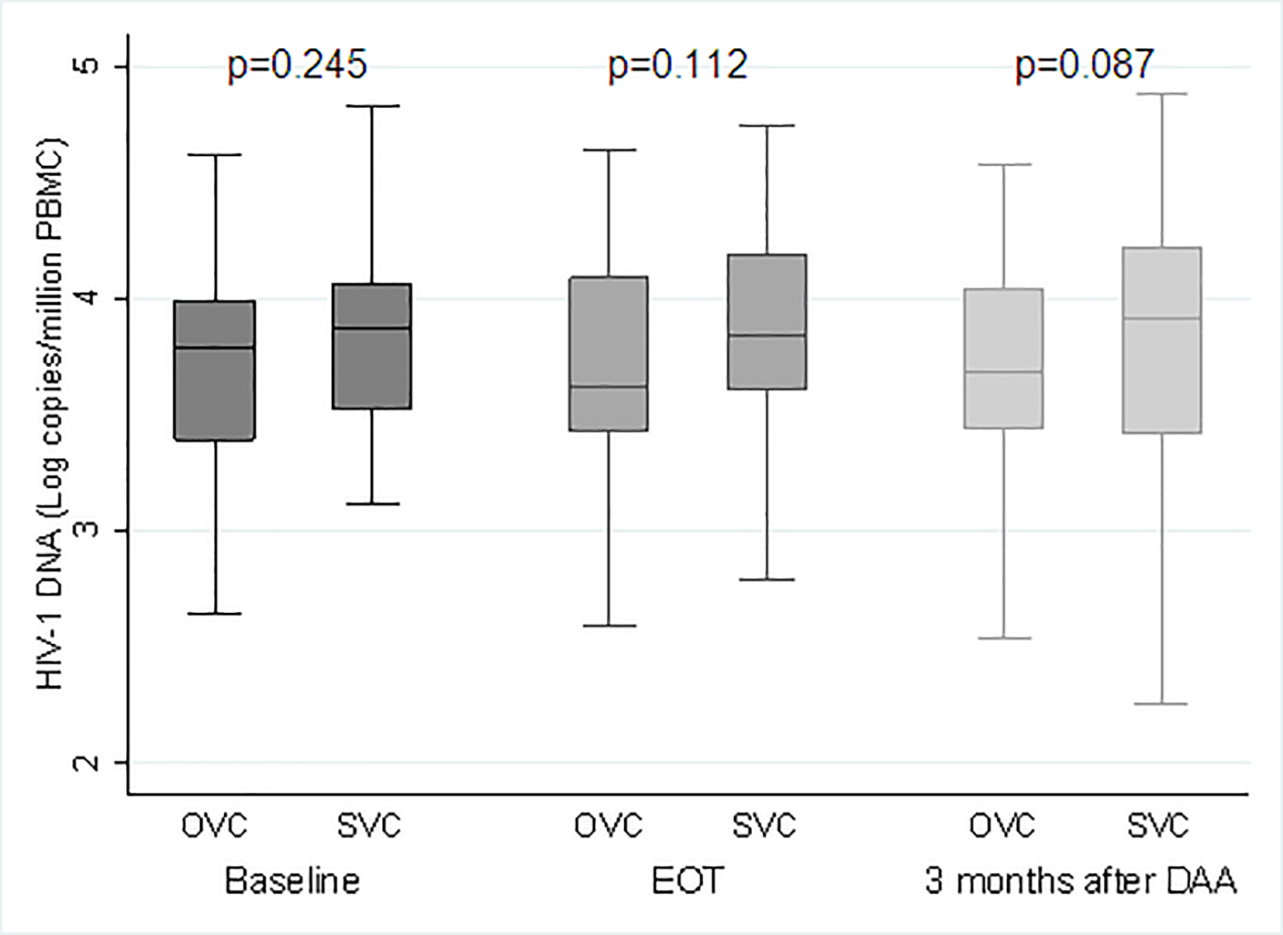
Median PBMC-associated total HIV-1 DNA at baseline, EOT and 3 months after DAA in OVC and SVC.

Median total HIV-1 DNA at baseline was 3.84 Log copies/million PBMC (95%CI, 3.49–4.05), with no significant variation over time: 3.78 Log copies/million PBMC (95%CI, 3.51–4.10) at EOT (p = 0.688), and 3.77 Log copies/million PBMC after 3 months of DAA (95%CI, 3.43–4.15) (p = 0.521). In Fig 1, median HIV-1 DNA values at baseline, at EOT and after 3 months of DAA completion are shown in OVC (n = 39, 40.2%) and SVC (n = 58, 59.8%). No significant difference between median baseline HIV-1 DNA value and any follow-up time-points was found in the two groups of patients.

More in depth, when comparing median values of total HIV-1 DNA at baseline and at EOT in 97 patients, 16 cases (16.7%) had an HIV-1 DNA increase ≥0.5 Log copies/million PBMC. In this subgroup of patients, the median change was higher from baseline to EOT (+0.62 Log copies/million PBMC [IQR, 0.56–0.84]), than from baseline to 3 months after DAA (+0.41 Log copies/million PBMC [IQR, 0.16–0.56]). Comparison of median HIV-1 DNA values at the two time-points in respect to baseline showed a statistically significant difference at EOT (3.54 Log copies/million PBMC *versus* 4.23 at EOT, p<0.001) and after 3 months of DAA termination (3.61 Log copies/million PBMC *versus* 3.84 at 3 months after DAA, p = 0.002). Among patients with HIV-1 DNA increase, 7/16 (43.9%) were OVC and 9/16 (56.3%) were SVC. No significant difference in frequencies of OVC and SVC were found between the group of patients with HIV-1 DNA increase and the remaining patients (p = 0.785). No significant difference in frequency of patients with HIV-1 DNA increase was found among those with or without HCV relapse (0% *versus* 17.6%; p = 0.584).

A counterbalancing subgroup of 12 patients (12.4%) showed a reduction of ≥0.5 Log copies/million PBMC with a median of -0.77 Log copies/million PBMC (IQR, -0.82-(-0.66)).

Identification of factors possibly associated with an increase of HIV-1 DNA (>0.5 Log/million PBMC from baseline to EOT) are shown in Table 2. At univariable analysis, a significantly higher risk of HIV-1 DNA increase was associated with lower baseline HIV-1 DNA levels (p = 0.006). Furthermore, higher duration of HIV suppression, change of ARV drugs before start of DAA, treatment with raltegravir-based ART, and previous exposure to interferon/ribavirin showed a significantly higher risk (p<0.05). At multivariable analysis, an independent effect was found for lower baseline HIV-1 DNA levels, longer duration of HIV suppression, treatment with raltegravir and previous exposure to interferon/ribavirin.

**Table 2:** Factors associated with increase of HIV-1 DNA (≥0.5 log_10_ copies/million PBMC) at EOT when compared to baseline at univariable and multivariable analysis.

		Univariable			Multivariable			
	OR	95%CI		p	OR	95%CI		p
Age (per each 10-years older)	2.70	0.99	7.35	0.052				
Gender								
- male	1.00							
- female	0.63	0.19	2.05	0.441				
Years of HIV infection (per each 1-year more)	1.01	0.94	1.09	0.679				
Plasma baseline HIV-1 RNA								
- detectable <40 copies/mL	1.00							
- not detectable <40 copies/mL	0.74	0.21	2.60	0.636				
Total baseline HIV-1 DNA (per each Log_10_ copies/million PBMC more)	0.20	0.06	0.62	0.006	0.20	0.07	0.58	0.003
Years of HIV suppression (per each 5-years more)	2.37	1.20	4.70	0.013	1.23	1.04	1.44	0.014
CD4 cell count at T0 (per each 100 cells/mmc more)	1.15	0.99	1.33	0.071				
CD4/CD8 ratio at T0								
- <1	1.00							
- ≥1	2.68	0.90	8.02	0.078				
Change in total lymphocytes between T0 and EOT (per each 100 cells/mmc more)	0.99	0.94	1.04	0.717				
Change in percentage of CD4 (per each 5% more)	0.96	0.47	1.98	0.922				
Change of ART before DAA	3.13	1.04	9.41	0.042	1.04	0.20	5.15	0.960
INSTI-based ART	3.24	1.08	9.73	0.036				
Raltegravir-including ART	3.50	1.15	10.63	0.027	8.36	1.40	50.02	0.020
HIV-1 RNA suppression during DAA								
- OVC	1.00							
- SVC	0.83	0.16	4.17	0.824				
Baseline HCV RNA (per each Log more)	2.01	0.93	4.39	0.078				
Cirrhosis								
- no	1.00							
- yes	0.48	0.16	1.50	0.209				
Fibrosis staging								
- F2	1.00							
- F3/F4	0.38	0.11	1.30	0.123				
IL28 B subtypes								
- CC	1.00							
- CT	0.64	0.09	4.53	0.657				
- TT	1.50	0.20	11.24	0.693				
Previous exposure to IFN/RBV for HCV treatment	3.39	1.11	10.30	0.031	5.17	1.18	22.61	0.029
Response to previous HCV treatment								
- no treatment	1.00							
- no response	3.22	0.80	12.94	0.099				
- partial response	1.81	0.18	18.31	0.614				
- relapser	2.72	0.60	12.42	0.197				
Type of DAA regimen								
- 2D/3D	1.00							
- sofosbuvir-based	0.47	0.10	2.24	0.340				
Duration of DAA treatment								
- 12 weeks	1.00							
- 24 weeks	1.45	0.49	4.28	0.496				
HCV RNA of <12 IU/mL at month 1 after start of DAA	0.68	0.23	2.02	0.486				

ART = antiretroviral therapy; DAA = directly acting antivirals.

**Fig 2:**
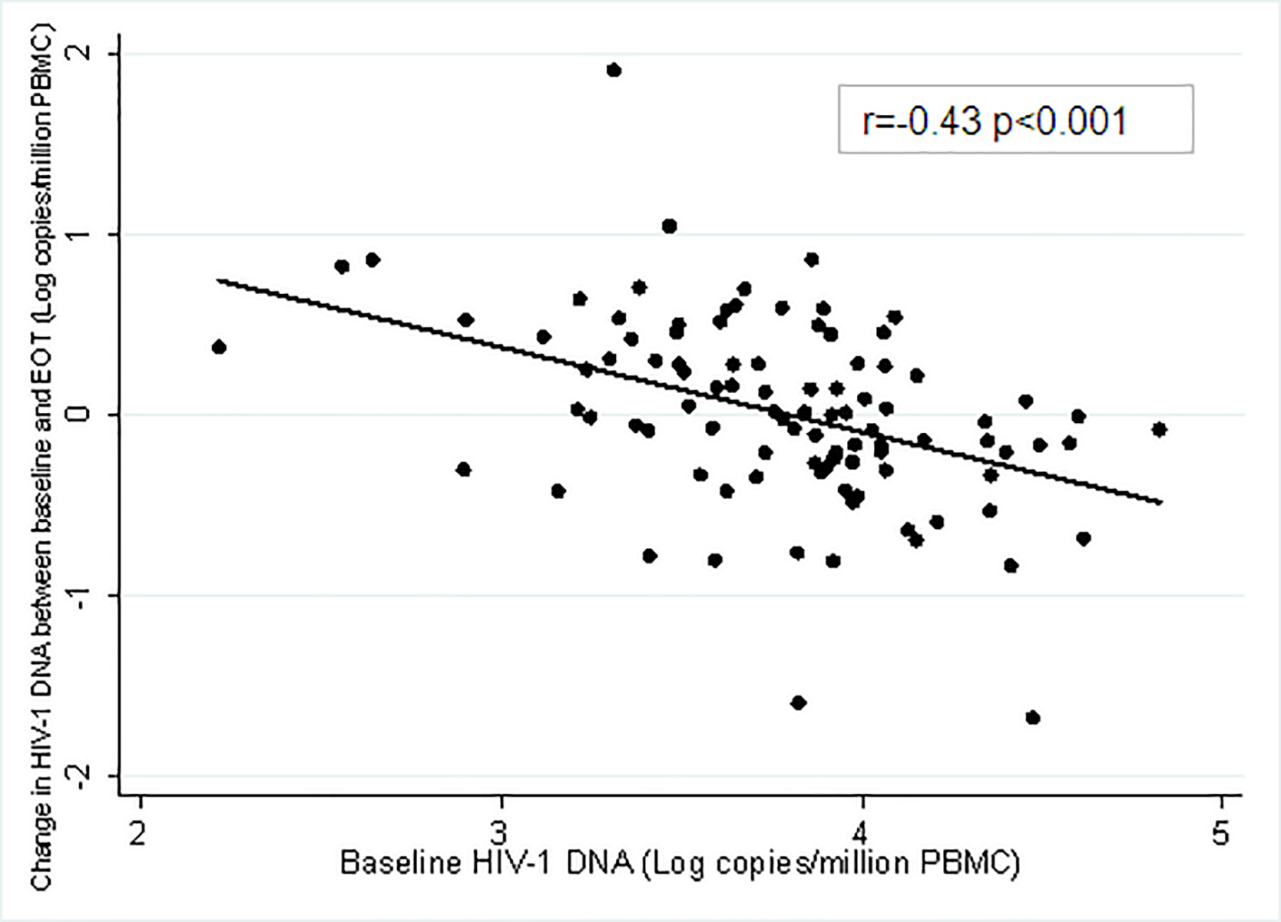
The scatterplot of baseline HIV-1 DNA levels and change between EOT and baseline.

Fig 2 shows the correlation of the baseline total HIV-1 DNA and its change between EOT and baseline: higher baseline HIV-1 DNA levels inversely correlated with HIV-1 DNA change (r = -0.43; p<0.001).

### Episomal 2-LTR HIV-1 DNA, residual plasma viremia, viral quasispecies and immunological variables

Additional explanatory virological examinations were performed focusing on specific subgroups of patients. In 14/16 patients with HIV-1 DNA increase ≥0.5 Log copies/million PBMC at EOT compared to baseline and in a control group of 34/81 subjects without HIV-1 DNA increase, unintegrated 2-LTR HIV-1 DNA episomal forms were assessed with a mean detectability threshold of 32 copies/million PBMC. At T0, 2-LTRs were below detectability threshold in all the 14 patients (100%) with HIV-1 DNA increase, whereas in the control group 2-LTR forms were detected in 5 out 34 patients (14.7%), representing on average the 61.3% of total HIV-1 DNA. At EOT, 2-LTR forms were detected in 2 cases (14.3%) with HIV-1 DNA increase (on average 23% of total HIV-1 DNA), and in 5 patients (14.7%) of the control group, representing on average the 49.0% of total HIV-1 DNA. All except one of these 5 patients were different from those with detectable forms at T0. The comparison of 2-LTR frequencies between patients experiencing HIV-1 DNA increase and the control group was not statistically significant at both baseline and EOT.

In the 6/7 OVC with HIV-1 DNA increase, residual HIV viremia <40 copies/ml was also investigated. Results indicated that residual viremia (detected in 2 patients at T0 and EOT, and in other 2 patients only at EOT) was not correlated with the detection of episomal 2-LTR HIV-1 DNA.

**Fig 3:**
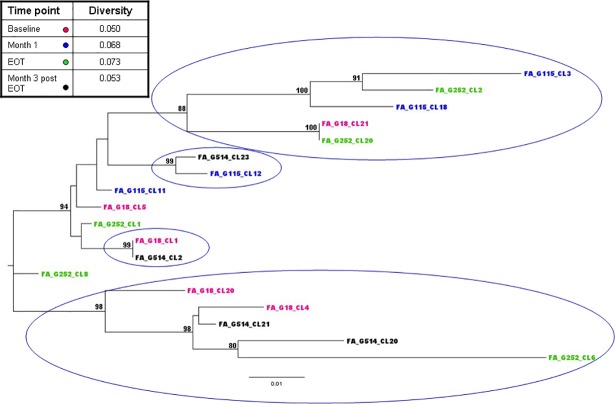
Phylogenetic tree of HIV-1 C2V5 nucleotide clonal sequences of subject #53 during DAA therapy. The viral sequences at the various time points are indicated by different colours, see table insert. Bootstrap values ≥80% are indicated. Bars indicate p distance scale. In the insert mean diversity (number of nucleotide substitutions/site) of the sequences detected at different time points is reported.

Viral quasispecies of proviral DNA was also analysed at different time points to verify possible viral evolution during DAA treatment, as possible consequence of new rounds of HIV-1 replications. For this purpose, 3 representative patients were studied: #53 (an OVC); #52 (a SVC with increase of total HIV-1 DNA); #43 (a SVC without total HIV-1 DNA increase). Results indicated that no statistically relevant increase of proviral quasispecies diversity occurred between T0 and follow-up times (i.e. T0, month 1, month 3, and month 6, p>0.05 for all the comparisons). In addition, to investigate genetic relationships among variants detected during DAA treatment, a phylogenetic tree for subject #53 with all sequences present at the different time points was constructed (Fig 3). Four distinct clusters of sequences were identified and each of them included variants belonging to different time points, suggesting that no viral evolution occurred in this patient during DAA.

Finally, in all patients showing an increase of total HIV-1 DNA during DAA, immunological parameters, such as total lymphocyte count, median CD4 cell count and CD4/CD8 ratio, were stable over time (i.e. at T0, month 1, month 3 and month 6, p >0.05 for all the comparisons) (data not shown).

## Discussion

In peripheral blood, cell-associated total HIV-1 DNA was quite stable over time in successfully ART-treated patients with HIV/HCV co-infection receiving DAA treatment. However, in about 17% of cases, a considerable increase in HIV-1 DNA was found at EOT, when compared to values found before start of DAA. Notably, this increase at EOT was more pronounced, than that observed between baseline and 3 months after DAA completion and was not related to the worsening of immunological parameters during DAA. Factors independently associated with this increase at EOT, were lower baseline HIV-1 DNA levels, longer HIV suppression, raltegravir-based ART and previous exposure to interferon/ribavirin for HCV treatment. The relationship between baseline HIV-1 DNA levels and its increase from baseline to EOT was confirmed by the significant inverse correlation between the two variables.

The findings reported in the present study add some interesting information to the topic of virological control of HIV infection during current HCV treatments and are consistent with the very scarce literature on this issue. The here reported HIV DNA increase in some HIV/HCV co-infected patients treated with DAA, is consistent with a recently published longitudinal study conducted in HIV/HCV co-infected patients with successful HCV treatment, where the extent of the phenomenon was greater in patients with undetectable HIV-1 RNA in plasma when compared to patients with low-level viremia during the previous year [19]. The possible interpretations for the here reported findings need to be discussed in the light of several hypothesis: first, raltegravir-based ART, often administered during DAA treatment, may have blocked HIV integration allowing for an increase of total HIV-1 DNA mostly made of episomal non-integrated forms. This thesis may be supported by the fact that to receive raltegravir-including ART was associated with a significantly higher risk of HIV DNA increase, but quantification of unintegrated episomal 2-LTRs at virological examinations was stable in patients with PBMC-associated total HIV-1 DNA growth. However, the effect of raltegravir on viral integration and consequently on 2-LTR forms accumulation it has been reported to be maximum during the first weeks of therapy [32], and our population was already receiving the antiretroviral for a longer period at the time of starting DAA. Secondly, virological analyses, performed to investigate active low-level HIV-1 replication, failed in finding an association between increased PBMC-associated total HIV-1 DNA and residual HIV-1 RNA measured with ultrasensitive assay, as well as no evidence of viral evolution was documented. Thirdly, the increase of HIV-1 DNA during DAA treatment in HIV/HCV co-infected patients could be explained by HIV-1 mobilization from reservoirs. It is well known that the lymph nodes, the liver and several other tissues [33,34] are HIV reservoirs, and it may be possible that during DAA treatment the virus is released from the organs to the peripheral blood. The effect of DAA on reservoirs may be a direct action on liver tissue or an indirect action through reduced HCV-driven immune activation [35] for the sudden fall of HCV viremia, which generally is observed during the first weeks of DAA therapy. Indeed, immune activation is one of the leading forces that keep lymphocytes sequestered in lymph nodes or in other tissues [36]. This hypothesis of the virus release from the reservoirs may also explain our finding of a more relevant PBMC-associated total HIV-1 DNA increase in patients with lower baseline HIV-1 DNA levels or longer duration of HIV suppression in the blood: in fact it is possible that HIV redistribution from reservoirs to the periphery may be more pronounced in patients with a better control of the infection in the blood.

To this regard is to mention the possible effect of IFN/RBV administrated as treatment for HCV infection in HIV/HCV co-infected patients in limiting HIV expression and replication, as already shown in [13–18].

Even if our study has some limitations, it may represent the basis for future research. Regrettably, blood samples were not available for all patients at all time points, but the overall number of analysed samples and the longitudinal fashion of the study contribute to the consistency of our findings. Further, evaluation of concomitant immune activation in plasma, characterization of the CD4 cell phenotype carrying proviral HIV-1 DNA in the peripheral blood, viral out-growth studies, and investigation of host genetic variations would have added relevant information regarding the complex interplay of viro-immunological parameters. Finally, extension of the follow-up after DAA treatment could be useful to evaluate long-term virological effects.

In conclusion, peripheral blood HIV-1 DNA in successfully ART-treated HIV/HCV co-infected patients undergoing DAA treatment was relatively stable over time, but some patients had a considerable increase of total HIV-1 DNA at EOT when compared to baseline. A significantly higher risk of HIV DNA increase was found, in presence of lower cellular HIV reservoir at baseline. Activation of replicative-competent virus generating new rounds of viral replication seems unlikely, while mobilization of HIV from tissue reservoirs, such as lymph nodes and the liver, could be hypothesized.
